# Identification of an Exosome-Related Signature for Predicting Prognosis, Immunotherapy Efficacy, and Tumor Microenvironment in Lung Adenocarcinoma

**DOI:** 10.1155/2022/1827987

**Published:** 2022-08-03

**Authors:** Tao Lin, Hong Wang, Xi He

**Affiliations:** ^1^Department of Thoracic Surgery, Tangshan People's Hospital, Tangshan, China; ^2^Tangshan Central Blood Station, Tangshan, China

## Abstract

Accumulating evidence suggests that exosomes can affect lung adenocarcinoma (LUAD) progression. However, there is still a lack of understanding of the global influence of exosome-related genes (ERGs) on prognostic relevance, tumor microenvironment features, and immunotherapy responsiveness in patients with LUAD. In the TCGA dataset, differential analysis of 490 LUAD samples and 59 normal samples yielded 30 ERGs with differential expression. We have created a predictive signature based on 10 overall survival (OS)-related ERGs and confirmed it in two external cohorts (GSE72094 and GSE68465) via the least absolute shrinkage and selection operator (LASSO) and Cox regression analysis in the TCGA dataset. The new signature revealed superior robustness and prognostic capacity for overall patient survival. Univariate and multivariate Cox regression analyses indicated that this signature was an independent risk factor for survival in patients with LUAD. In addition, for predicting the 1-year, 3-year, and 5-year OS of LUAD patients, we developed a nomogram and confirmed its predictive ability via the C-index and calibration curve. In addition, patients categorized by risk score exhibited distinct immunological states, stemness index, immune subtypes, and immunotherapy response. In conclusion, we created a risk signature for LUAD that was tightly associated with the immune landscape and therapeutic response. Also, such a risk signature effectively promotes the ability of the clinicians in making more precise and individualized treatment recommendations for patients with LUAD.

## 1. Introduction

Lung cancer ranks second and first, respectively, in incidence (11.4%) and mortality (18.0%) among malignant tumors [1], with 2.2 million new cases and 1.8 million deaths globally in 2020. Lung adenocarcinoma (LUAD) is one of the major pathological subtypes of non-small cell lung cancer (NSCLC) [2]. Patients with LUAD lack tumor specificity and clinical symptoms in early stages, and local infiltration and even distant metastases occur in mid-to-late stage LUAD, with a poor outcome [3]. Despite recent advancements in molecularly targeted treatments and immunotherapy, improving patients' overall survival (OS) and progression-free survival remains a significant therapeutic challenge [4, 5]. The development of new valuable biomarkers has important clinical implications for prognosis prediction and individualized treatment.

Exosomes are small vesicles, usually 40–100 nm in diameter, that are secreted outward by cells [6]. Exosomes contain proteins, lipids, RNA, and other substances, which can be received by recipient cells to achieve material transport and information transfer between cells [7]. As an element widely present and distributed in various body fluids, the exosomes have the features, such as, carrying and transmitting important signaling molecules, forming a new cell-cell information transfer system, influencing the physiological state of cells, and actively participating in a variety of biological processes including immune response, antigen presentation, and cell differentiation [8, 9]. Increasing evidence indicates that exosomes secreted by tumor cells are involved in tumorigenesis, growth, invasion, and metastasis [10]. Tumor immunity is modulated by the communication provided by exosomes between immune cells and tumor cells. Exosomes can either trigger antitumor responses by suppressing immune cells (DCs, NK cells, CD4^+^ and CD8^+^ T cells, etc.) or induce immunosuppression or modulate immunosuppression of cell populations (MDSCs, Tregs, and Bregs) [11, 12].

In this investigation, ten ERGs were used to create a risk signature. Its prognostic value, diagnostic efficacy, immunotherapy efficacy, and tumor immune infiltration in LUAD patients were also investigated. It may provide a crucial foundation for future research.

## 2. Materials and Methods

### 2.1. Dataset and Preprocessing

Samples with no survival status and overall survival (OS) of <30 days were excluded. The RNA-Seq data of LUAD patients were downloaded from the TCGA database (in TPM format and log-transformed), and 490 tumor samples and 59 normal samples were included. The GSE72904 and GSE68465 datasets were downloaded from the GEO database, annotated with their respective platform files, and used as the validation cohorts. In the survival analysis, 490 patients were included in TCGA-LUAD, 386 in GSE72904, and 210 in GSE68465. The batch effect was removed for GSE72904, GSE68465, and TCGA-LUAD using the combat function in the “sva” package. GSE78220 and IMvigor are immunotherapy cohorts in which clinical information includes response to immunotherapy. In addition, 121 exosome-related genes (ERGs) were downloaded from the ExoBCD database (https://exobcd.liumwei.org/).

### 2.2. Construction and Validation of Prognostic Signature

First, we used the “limma” package to explore the DEGs (FDR <0.05, logFC >1) of 121 ERGs between normal and tumor samples (TCGA-LUAD cohort). The TCGA-LUAD cohort was used as the training set. The external validation set consists of GSE68465 and GSE72904 cohorts. By the adoption of the least absolute shrinkage and selection operator (LASSO) Cox regression, we removed the redundant genes in DEGs in theTCGA-LUAD cohort and then produced a risk score formula via the multivariable Cox regression. Patients were divided into high- and low-risk groups according to the median risk score. Univariate and multivariate cox regression analyses were used to assess the prognostic value of risk score in both the training set and the external validation set. In addition, we evaluated the prognostic performance of the risk signature by employing the Kaplan–Meier and time-dependent receiver operating characteristic (ROC) analyses. A prognostic nomogram was constructed using independent prognostic factors identified by multivariate Cox regression and validated using the calibration curve.

### 2.3. Immune Analysis

Different methods, including TIMER, CIBERSORT, QUANTISEQ, MCP-counter, XCELL, and EPIC, were employed concurrently to estimate the immune cell infiltration in different samples during immune cell analysis. Using the ESTIMATE algorithm, stromal score, ESTIMATE score, and tumor purity were calculated to characterize the condition of the tumor microenvironment. Thorsson et al. [13] defined six immune expression signature subtypes based on the gene expression profiles of all solid tumors in TCGA, including wound healing (Immune C1), IFN-gamma dominant (Immune C2), inflammatory (Immune C3), lymphocyte depleted (Immune C4), immunologically quiet (Immune C5), and TGF-beta dominant (Immune C6).

## 3. Results

### 3.1. Construction of an Exosome Prognostic Signature in Training Set

**Figure 1:**
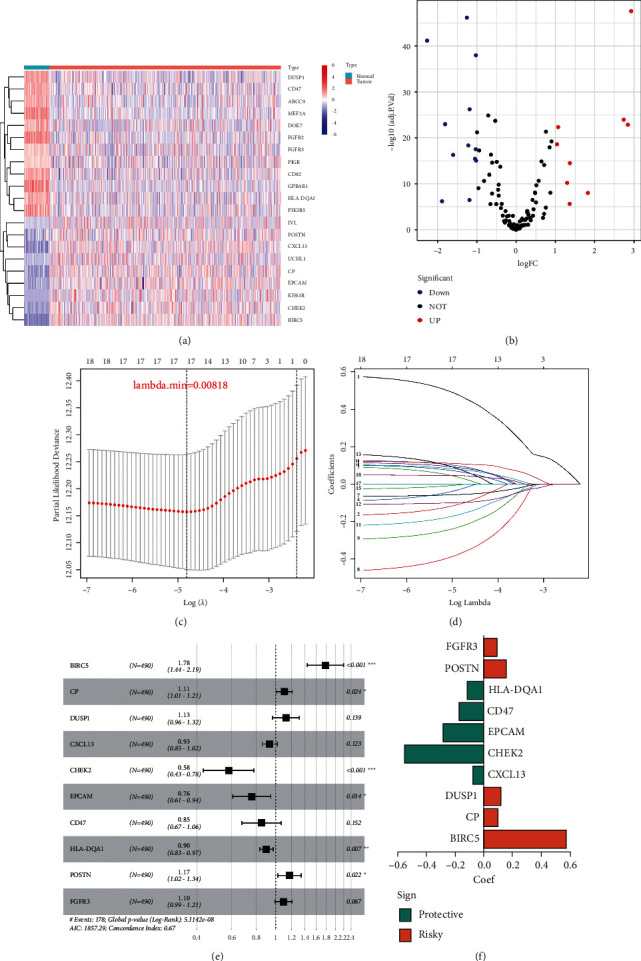
Construction of ERG prognostic signature in the training set. (a) The heat map indicated ERG expression in LUAD and normal samples. (b) The volcano plot exhibited both down- and upregulated ERGs. (c) Tuning parameter selection using cross-validation in the LASSO model. (d) The coefficient profile of LASSO for 17 ERGs. (e) Multivariate Cox regression analysis of ERGs.

We discovered 30 differentially expressed ERGs through differential analysis of 490 LUAD cases and 59 normal samples, comprising 9 upregulated and 12 downregulated genes (Figures 1(a) and 1(b)). To construct a prognostic prediction signature for patients with LUAD, we performed LASSO regression analysis on 30 differentially expressed ERGs in the training set and determined a minimum lamba value of 0.00818, retaining 17 genes (Figures 1(c) and 1(d)). Subsequently, multivariate Cox regression analysis was used for rescreening and 10 genes were finally included for the construction of prognostic signature (Figure 1(e)). To quantify the risk score, we placed the above genes in a Cox regression equation to obtain correlation coefficients (Figure 1(f)). Risk score = (0.5756^*∗*^BIRC5) + (0.1019^*∗*^CP) + (0.1179^*∗*^DUSP1) + (−0.0694^*∗*^CXCL13) + (−0.5461 ^*∗*^CHEK2) + (−0.2781^*∗*^EPCAM) +(−0.1662^*∗*^CD47) + (−0.1077^*∗*^HLA-DQA1) + (0.15833^*∗*^POSTN) + (0.0911^*∗*^ FGFR3). Using the median risk score as the dividing line, we classified all LUAD patients in the training cohort and the external validation cohort (GSE72094 or GSE68465) into two subgroups: high-risk and low-risk.

### 3.2. Validation of the Exosome-Related Prognostic Signature

**Figure 2:**
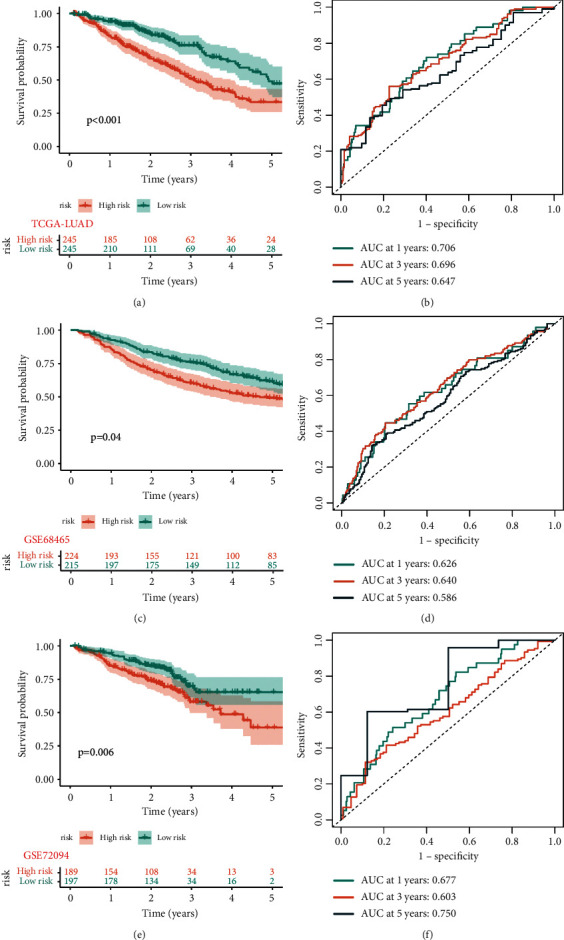
Validation of the prognostic signature for LUAD patients in the testing set and two external validation sets. Kaplan–Meier curves showed that the high-risk group had worse overall survival (OS) than the low-risk group in the testing set (a), GSE68465 set (c), and GSE72094 set (e). ROC curves and their AUC values represented 1-, 3-, and 5-year predictions in the testing set (b), GSE68465 set (d), and GSE72094 set (f).

To verify the predictive power of prognostic signature, we performed survival analysis and ROC analysis of LUAD patients from different cohorts based on the risk score. In the training set, Kaplan–Meier analysis revealed that the survival rate of LUAD patients in the high-risk subgroup was considerably lower than that of patients in the low-risk subgroup (*p* < 0.001). Figures 2(a) and 2(b) depict ROC curves indicating that the prognostic signature showed a significant predictive value for LUAD patients in the testing set (1-year AUC = 0.706, 3-year AUC = 0.696, and 5-year AUC = 0.647). Moreover, the prognostic signature demonstrated excellent prognostic significance for LUAD patients in the GSE68465 cohort (1-year AUC = 0.626, 3-year AUC = 0.640, and 5-year AUC = 0.586; Figure 2(d)) and the GSE72094 cohort (1-year AUC = 0.677, 3-year AUC = 0.603, and 5-year AUC = 0.750; Figure 2(e)). Kaplan–Meier survival curves revealed that the low-risk group had greater survival than the high-risk group (*p* < 0.05) (Figures 2(c) and 2(e)).

**Figure 3:**
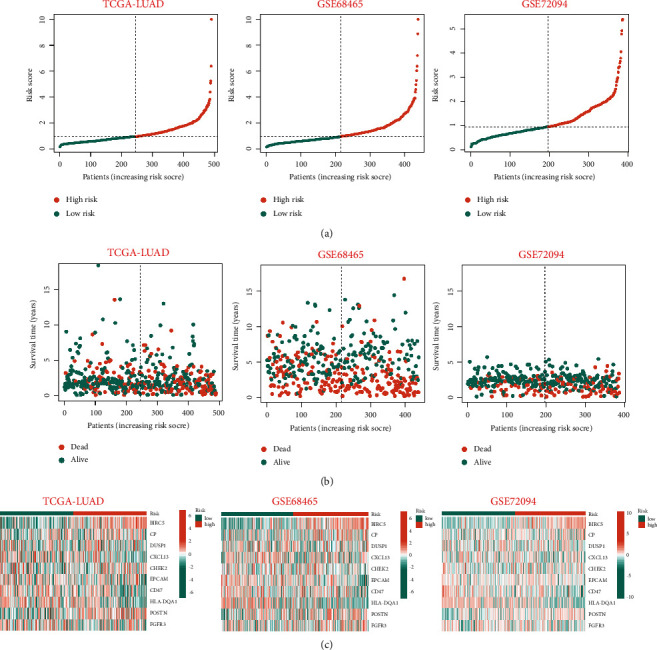
Evaluation and validation of the utility of prognostic signature in the training set and validation sets. (a) The risk score distribution plot showed the distribution of high-risk and low-risk LUAD patients in the testing set, GSE68465 set and GSE72094 set. (b) The scatter dot plot showed the outcomes between the survival status and risk score of LUAD patients in the high- and low-group. (c) Heatmap of the 10 ERGs expression profiles in the high-risk and low-risk group in the testing set and two external validation sets, separately.

**Figure 4:**
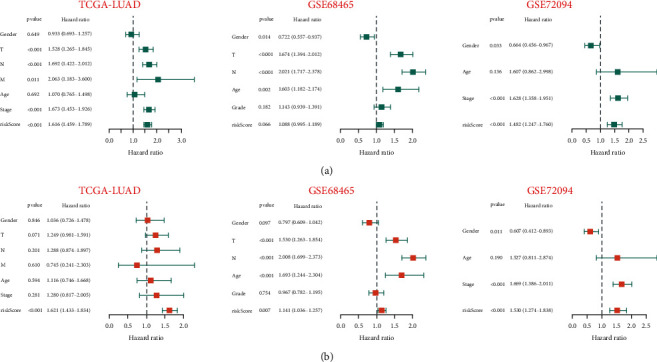
Univariate and multivariate Cox analyses for the signature-based risk score and other clinical features. (a) A univariate Cox regression analysis validated the signature-based risk score as a predictive factor for the TCGA-LUAD and GSE72094 cohorts. (b) A multivariable Cox regression analysis validated the signature-based risk score as an independent predictive factor for the training cohort and two external validation cohorts.

The distribution plot of risk score and survival status revealed that the number of LUAD patients with a status of deceased increased as the risk score in the training set rose. In validation sets, the low-risk group maintained its superior survival status and longer survival time from the training set (Figures 3(a) and 3(b)). In addition, a heatmap demonstrated that the expression of 10 ERGs varied significantly among LUAD patients with varying risk scores (Figure 3(c)). We hypothesized that the prognostic signature could function as an independent prognostic factor for LUAD patients. In order to confirm this hypothesis, univariate and multivariate Cox regression analyses were conducted. In univariate Cox analysis, the signature-based risk score was found to be substantially associated with OS (TCGA-LUAD : HR = 1.616, *p* < 0.001; GSE68465 : HR = 1.088, *p*=0.066; GSE72094 : HR = 1.482, *p* < 0.001) (Figure 4(a)). Moreover, multivariate Cox analysis revealed that the risk score remained an independent risk factor (TCGA-LUAD : HR = 1.621, *p* < 0.001; GSE68465 : HR = 1.141, *p*=0.007; GSE72094 : HR = 1.530, *p* < 0.001) (Figure 4(b)). Consequently, the signature was an independent risk factor that affected the survival of LUAD patients.

### 3.3. Construction of the Clinical Nomogram

**Figure 5:**
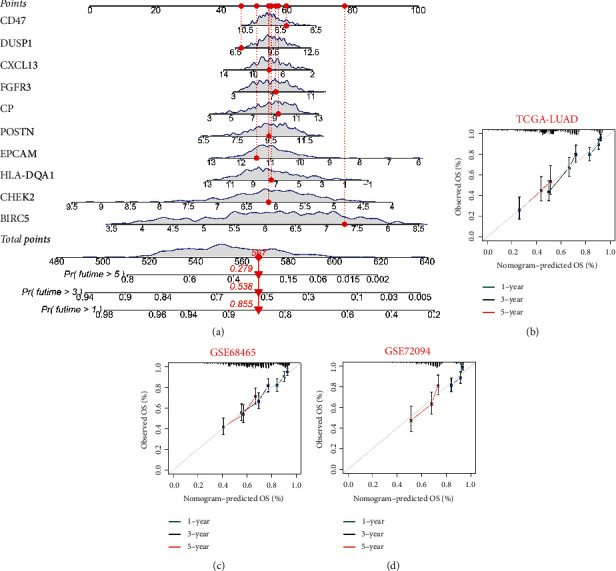
Construction and evaluation of the novel nomogram. (a) The nomogram for predicting the survival probability of LUAD patients. The calibration plots of the nomogram for predicting OS probability for 1, 3, and 5 years in the TCGA-LUAD cohort (B), GSE68465 cohort (c), and GSE72094 cohort (d).

Considering the complexity of the risk signature formula and the visual applicability of the nomogram in clinical work, we developed a nomogram to predict the 1-year, 3-year, and 5-year OS of LUAD patients (Figure 5(a)). In addition, the calibration curve revealed that the predicted curve was near to the true curve of LUAD patients, indicating that the predicted survival rate at 1, 3, and 5 years is closely related to the actual rates (Figures 5(b)–5(d)). The C-index of TCGA-LUAD, GSE68465, and GSE72094 were 0.823, 0.715, and 0.733, respectively. According to the preceding data, the nomogram is appropriate for clinically predicting the prognosis of LUAD patients.

### 3.4. Immune Characteristics in Different Risk Subgroups

**Figure 6:**
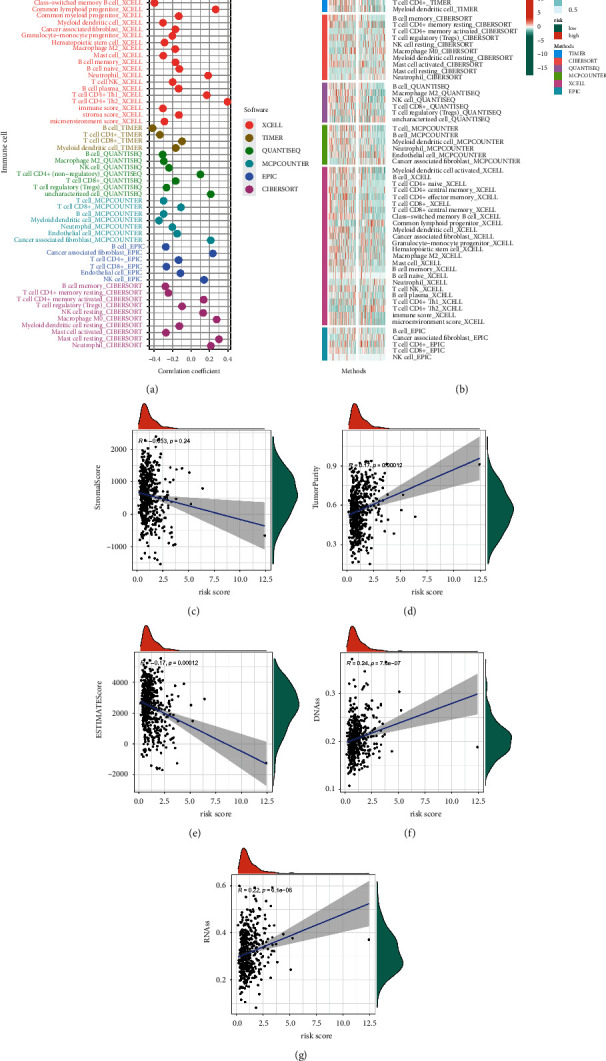
Immune characteristics in different risk subgroups. The correlation of tumor-infiltrating cells with risk score using 6 algorithms. (a) Heatmap. (b) lollipop plot. The correlation between risk score and the stromal score (c), tumor purity (d), and ESTIMATE score (e). (f, g) The correlation between risk score and stemness index.

To investigate the discriminative usefulness of the risk subgroup for TME and its application value in immunotherapy, we utilized six distinct algorithms to simultaneously evaluate the abundance of immune cell infiltration in various samples. Unsurprisingly, the number of killer immune cells (e.g., CD4^+^ T and CD8^+^ T cells) declined as the risk score grew, while the number of immunosuppressive cells (e.g., Treg) increased (Figures 6(a) and 6(b)). The association between the risk score and the TME score was then investigated. We concluded that the risk score is negatively linked to ESTIMATE and stromal scores and positively correlated with tumor purity (Figures 6(c)–6(e)). In light of the significant influence of the stemness index on immunotherapy, correlation research revealed that the risk score is positively correlated with DNAss and RNAss (Figures 6(f)–6(g)). These results demonstrate an immune activation status in the low-risk subgroup, which may benefit from immunotherapy.

### 3.5. Exosome Risk Signature Predicts Immunotherapy Outcomes

**Figure 7:**
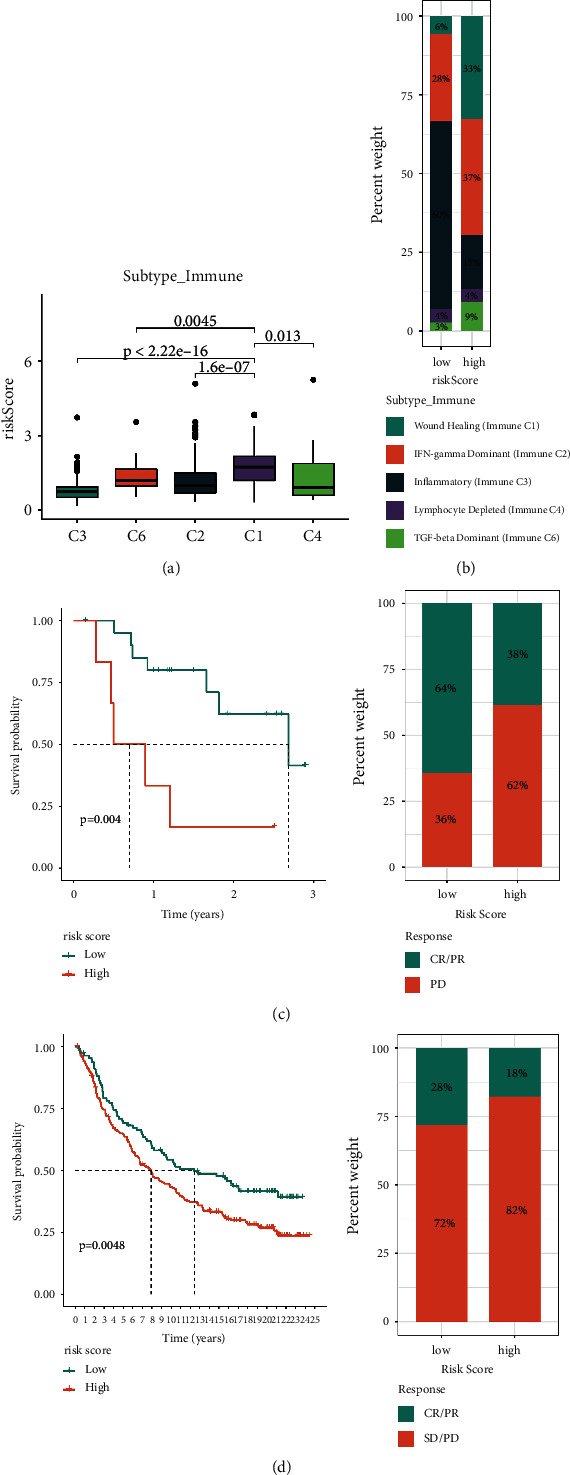
Association of risk subgroup with therapy in LUAD patients. (a, b) Relationships between risk score and immune subtypes. (c, d) The survival status and immunotherapy reflection of patients in the high- and low-risk subgroups in two immunotherapy cohorts. CR, complete response; PR, partial response; SD, stable disease; PD, progressive disease.

The C3 subtype had the lowest risk score among the six known immunological characteristic subtypes, whereas the C1 subtype had the greatest risk score (Figure 7(a)). In the low-risk grouping, C3 was more prevalent (Figure 7(b)). To validate the value of the risk score for survival prediction and treatment reflection prediction in the immunotherapy cohort, we performed validation in the two immunotherapy cohorts separately. Kaplan–Meier analysis revealed that patients undergoing immune treatment with a low-risk score had better OS. The percentage of responsive patients was higher in the low-risk score group (Figures 7(c) and 7(d)).

## 4. Discussion

Our study is, to the best of our knowledge, the first complete and detailed examination of ERGs in LUAD, which may serve as an important foundation for future research. First, we retrieved 30 differentially expressed ERGs from differential analysis of 490 LUAD samples and 59 normal samples from the TCGA database, including 9 upregulated genes and 12 downregulated genes. To develop a prognostic prediction signature for patients with LUAD, we conducted LASSO regression analysis on 30 differentially expressed ERGs in the training set and found a minimal lambda value of 0.00818 while retaining 17 genes. Subsequently, multivariate Cox regression analysis was utilized for rescreening, and ten genes were subsequently incorporated in the building of the prognostic signature.

We then developed a 10-gene risk signature to investigate the association between LUAD and ERGs. Updates confirm that several of these identified genes may play distinct functional functions in the progression of cancer. Specifically, BIRC5, CP, EPCAM, CXCL13, POSTN, HLA-DQA1, and CHEK2 have a high predictive value for LUAD [14–20]. As discovered by Yang and others, coexpression of PD-L1 and CD47 predicts survival and illuminates future dual-targeting immunotherapy in non-small cell lung cancer [21]. Jing et al. [22] found that miR-24-3p/FGFR3 signaling as a novel axis is involved in EMT and regulates lung adenocarcinoma progression.

In addition to determining the best cutoff point that discriminated the high- or low-risk group among patients with LUAD in the training cohort and the external validation cohort (GSE72094 and GSE68465), we performed Kaplan–Meier analysis and ROC to validate the prediction capacity of the risk signature. Using Kaplan–Meier analysis, it was evident that patients in the high-risk group had a considerable disadvantage in terms of survival. Surprisingly, we discovered that the training cohort and the two external validation cohorts exhibited similar consistency. In addition, ROC curves revealed that the prognostic signature had a high predictive value for LUAD patients in the testing set and two external sets (GSE72094 and GSE68465). In addition, a nomogram was developed to predict the 1-year, 3-year, and 5-year OS of LUAD patients, and its predictive ability was confirmed using the C-index and calibration curve.

Immune infiltration research revealed a negative association between the risk score and the infiltration of killer immune cells (such as CD4^+^ T and CD8^+^ T cells) and a positive correlation with the infiltration of immunosuppressive cells (such as Treg). In a recent study, CD4^+^ T cells were proven to be more recruited by BLCA cells, which promoted LUAD metastasis [23]. IL-9-producing tumor-infiltrating lymphocytes and Treg subsets drive immune escape of tumor cells in non-small cell lung cancer [24]. Using the gene expression profiles of all solid tumors in TCGA, Thorsson et al. [13] identified six immune expression signature subtypes. We discovered that the expression of risk score was lowest in the C3 subtype and highest in the C1/C6 subtypes, while the low-risk subgroup had a higher proportion of C3. In light of the significant impact of stemness index on immunotherapy, correlation analysis revealed a positive relationship between risk score and DNAss and RNAss. All of the aforementioned findings revealed that patients with low-risk scores have a highly activated immune system, which may have a good effect on immunotherapy. Comprehensive validation analysis of two immunotherapy datasets (GSE78220 and IMvigor) revealed that patients with a low-risk score who were treated with immunotherapy had improved OS and immunotherapy response. All of the aforementioned data suggested that our signature may be a suitable index for evaluating the immunotherapy response in patients with LUAD.

## 5. Conclusion

Overall, our study indicated that exosomes are strongly associated with LUAD. In addition, the risk signature formed from 10 ERGs may provide a novel method for accurate clinical outcome prediction and selection of individualized treatment strategies.

## Data Availability

The data are available at the TCGA (https://portal.gdc.cancer.gov/) and GEO (https://www.ncbi.nlm.nih.gov/geo/).
